# Reproducibility of each retinal layer thickness measurement in epiretinal membrane patients with ectopic inner foveal layers

**DOI:** 10.1186/s40662-022-00321-2

**Published:** 2023-01-04

**Authors:** Il Jung, Yong-Jin Na, Sung-Chul Lee, Min-Woo Lee

**Affiliations:** 1 Department of Ophthalmology, Konyang University College of Medicine, #1643 Gwanjeo-Dong, Seo-Gu, Daejeon, Republic of Korea; 2 Konyang University Myunggok Medical Research Institute, Daejeon, Republic of Korea

**Keywords:** Epiretinal membrane, Ectopic inner foveal layer, Inner retinal layer

## Abstract

**Background:**

To identify the reliability of each retinal layer thickness measurement in epiretinal membrane (ERM) patients with ectopic inner foveal layers (EIFLs).

**Methods:**

Subjects were divided into two groups: ERM patients with EIFLs (Group 1) and without EIFLs (Group 2). The retinal layer thickness was measured twice, and intraclass correlation coefficient (ICC) and coefficient of variation (CV) values were calculated.

**Results:**

In Group 1, the CVs of the nerve fiber layer (NFL), ganglion cell layer (GCL), inner plexiform layer (IPL), inner nuclear layer (INL), outer plexiform layer (OPL), and outer nuclear layer (ONL) were 22.39%, 13.12%, 13.37%, 13.21%, 15.09%, and 11.94%, while the ICCs were 0.431, 0.550, 0.440, 0.286, 0.279, and 0.503, respectively. In Group 2, the CVs were 18.20%, 10.59%, 10.65%, 13.27%, 14.75%, and 10.37%, while the ICCs were 0.788, 0.834, 0.830, 0.715, 0.226, and 0.439, respectively. The average central macular thickness (CMT) was significantly correlated with the CVs of NFL (coefficient = 0.317; *P* < 0.001), GCL (coefficient = 0.328; *P* < 0.001), and IPL (coefficient = 0.186; *P* = 0.042) in Group 1.

**Conclusions:**

The reproducibility of the inner retinal layer thickness measurements in ERM patients with EIFLs was low compared to those without EIFLs. The reproducibility of the outer retinal layer thickness measurements, including OPL and ONL, was poor regardless of the presence of EIFLs in ERM patients. Additionally, the thicker the CMT in patients with EIFLs, the lower the reproducibility of the inner retinal layer thickness measurements.

## Background

Idiopathic epiretinal membrane (ERM) is a common disease affecting 7% of the population [1]. It is caused by fibrocellular proliferation on the inner retinal surface of the macular area which impairs visual acuity and leads to metamorphopsia through retinal tangential traction [2]. Surgical removal of ERM is the choice of treatment, and many prognostic factors have been reported, including central macular thickness (CMT), preoperative visual acuity, disruption of the cone outer segment tip line before surgery, symptom duration, preoperative inner retinal layer thickness, and the presence of an ectopic inner foveal layers (EIFLs) [3–7].

Among these, EIFLs are representative important factors associated with postoperative visual outcomes.  The EIFL, a continuous floor of inner retinal tissue extending from the inner nuclear layer (INL) and inner plexiform layer (IPL) across the central fovea, would be induced by the displacement and reorganization of the inner retinal layers due to the chronic anteroposterior and centripetal traction by the ERM [8]. Gonzalez et al. [9] reported that patients with continuous and clearly defined EIFL were less likely to show anatomical improvement. Moreover, despite the significant visual improvement, their final visual outcomes can be compromised. Govetto et al. [10] reported significantly lower postoperative visual and anatomical restoration in idiopathic ERM with EIFL. Additionally, the thickness of these abnormal inner retinal layers is another important prognostic factor. A previous study found that EIFL thickness had a strong negative correlation with both pre- and postoperative visual acuity [10]. Yang et al. [11] also reported that postoperative visual outcomes correlated well with preoperative central inner retinal layer thickness. Although preoperative measurement of inner retinal layer thickness is crucial in ERM patients, accurate measurement of each retinal layer thickness is difficult due to the irregular margins of the inner retinal layer caused by structural damage, especially in patients with EIFLs. It can then be assumed that the automatic measurement of each retinal layer thickness is inaccurate in patients with EIFLs. However, a detailed study analyzing the reliability of automatic measurement in patients with EIFLs has not been reported.

Repeatability and reproducibility, the degree of agreement between the results of measurements conducted by the same or different conditions, are crucial factors in the reliability of the test. Previous studies reported the reliability of optical coherence tomography (OCT) in healthy subjects [12–14]. In this study, we aimed to identify the reliability of automatic spectral-domain OCT (SD-OCT) measurements of each retinal layer thickness via analyses of short-term reproducibility in patients with EIFLs, compared to ERM patients without EIFLs.

## Methods

### Patients

This study followed the tenets of the Declaration of Helsinki and was approved by the Institutional Review Board/Ethics committee of Konyang University Hospital, Republic of Korea. We reviewed the charts of consecutive idiopathic ERM patients treated with pars plana vitrectomy at Konyang University between January 2018 and December 2021. We recorded detailed histories, best-corrected visual acuity (BCVA), intraocular pressure, spherical equivalent, and axial length. The requirement for informed consent was waived due to the retrospective nature of the study, which was approved by the Institutional Review Board/Ethics committee of Konyang University Hospital. EIFLs were defined on OCT images as the presence of a continuous hyporeflective or hyperreflective band, extending from INL and IPL across the foveal region and visible in all OCT scans centered on the fovea [8]. Subjects were divided into two groups: ERM patients showing EIFL on the OCT images (Group 1) and those without EIFL on the OCT images (Group 2). The exclusion criteria included the following: a history of ophthalmic diseases other than ERM, such as retinal detachment, inflammatory eye disorders, myopic schisis, vitreomacular traction syndrome, full-thickness macular hole, retinal vessel occlusion, glaucoma, high myopia with an axial length ≥ 26.0 mm, and previous intraocular surgery excluding cataract extraction. If both eyes met the inclusion criteria, the eye with the higher image quality was selected for analysis.

### OCT measurements

SD-OCT (Spectralis; Heidelberg Engineering, Heidelberg, Germany) was performed twice at intervals of less than one week before surgery by a skilled examiner, using the AutoRescan mode. We used a volume comprising at least 49 B-scans with a distance between B-scans of 120 μm over a 20° × 20° square centered on the fovea. Scans were acquired in high-resolution mode, using automatic real-time for averaging 16 B-scan frames to reduce noise and improve image quality. An internal fixation light was used to center the scanning area on the fovea. The foveal thicknesses obtained using SD-OCT were measured by the built-in Spectralis mapping software (Heidelberg Eye Explorer ver. 6.9a). We used retinal thickness map analyses to display numeric averages of the measurements for the fovea, the central circle with a 1-mm diameter of the Early Treatment Diabetic Retinopathy Study (ETDRS) subfield. Automated retinal layer segmentation was performed, and the thickness of each foveal layer was determined using the automated Spectralis segmentation software. The CMT and thicknesses of the retinal nerve fiber layer (NFL), ganglion cell layer (GCL), IPL, INL, outer plexiform layer (OPL), and outer nuclear layer (ONL) in the central circle of the ETDRS were obtained. Images with quality scores < 15 dB (range, 0 to 40 dB) or algorithm segmentation failure were excluded.

### Statistical analysis

Demographic characteristics and ocular parameters were compared using the independent t-test and Chi-squared test. The paired t-test was used to examine the difference between the two measurements in each group. The reproducibility of each retinal layer thickness measurement in the central circle of the ETDRS subfield was assessed using the intraclass correlation coefficient (ICC), coefficient of variation (CV), and reproducibility limit. ICC is the ratio of subject variance to the total variance, and a value close to 1 suggests low variance of the examination (poor: < 0.40; fair: 0.40–0.59; good: 0.60–0.74; excellent: 0.75–1.00). The CV (%) was calculated as 100 × standard deviation/overall mean, and a value of < 10% indicates good reproducibility [15–17]. The reproducibility limit was calculated as 1.96√2 × standard deviation, which represents the expected limits that 95% of the measurements should fall within. The agreement between the two measurements was evaluated using Bland–Altman plots. Pearson’s correlation analyses were performed for the CV of each retinal layer thickness and the mean CMT of the two measurements to identify the relationships between the reproducibility of each retinal layer thickness measurement and CMT. Linear regression analyses were performed to identify the association between the average CMT and difference between the two measurements in each retinal layer. All statistical analyses were performed using the SPSS Statistics software (ver. 18.0; IBM Corp., Armonk, NY, USA).

## Results

### Demographics

A total of 173 eyes were enrolled: 120 eyes for Group 1 and 53 eyes for Group 2. The mean ages in Group 1 and Group 2 were 67.0 ± 7.7 and 64.2 ± 7.8 years, respectively (*P* = 0.060), which was not significantly different (Table 1). Except for BCVA (0.46 ± 0.26 *vs.* − 0.36 ± 0.22 logMAR; *P* < 0.001), sex, laterality, spherical equivalent, intraocular pressure, and axial length were not significantly different between the two groups.

**Table 1:** Patient demographics

Parameter	Group 1 (n = 120)	Group 2 (n = 53)	*P* value
Age (years, mean ± SD)	67.0 ± 7.7	64.2 ± 7.8	0.060
Sex (male, %)	49 (40.8)	24 (45.3)	0.585
Diabetes (n, %))	105 (87.5)	44 (83.0)	0.432
Hypertension (n, %)	60 (50.0)	27 (50.9)	0.909
Laterality (right, %)	61 (50.8)	28 (52.8)	0.809
BCVA (logMAR, mean ± SD)	0.46 ± 0.26	− 0.36 ± 0.22	** < 0.001**
Intraocular pressure (mmHg, mean ± SD)	13.9 ± 3.2	14.2 ± 2.8	0.494
Spherical equivalent (D, mean ± SD)	0.03 ± 1.56	0.19 ± 1.76	0.571
Axial length (mm, mean ± SD)	23.5 ± 0.9	23.7 ± 0.9	0.131

*BCVA* = best-corrected visual acuity

Bold values indicate statistically significant (*P* < 0.050)

### OCT measurement

In Group 1, the first thickness measurements of CMT, NFL, GCL, IPL, INL, OPL, and ONL were not significantly different from the second measurements (Table 2).

**Table 2:** Measurements of each retinal layer

Parameter	Group 1			Group 2		
	First (μm)	Second (μm)	*P* value	First (μm)	Second (μm)	*P* value
CMT	494.8 ± 70.2	497.5 ± 71.5	0.119	402.7 ± 69.2	401.1 ± 65.9	0.528
NFL	106.4 ± 76.6	110.0 ± 80.5	0.257	36.8 ± 42.7	34.5 ± 41.2	0.545
GCL	50.1 ± 11.8	50.8 ± 12.0	0.503	43.2 ± 14.3	44.4 ± 15.7	0.323
IPL	49.0 ± 10.3	49.4 ± 11.1	0.694	37.8 ± 12.3	39.5 ± 12.2	0.104
INL	53.9 ± 10.2	52.9 ± 9.7	0.355	47.6 ± 14.2	46.8 ± 13.2	0.563
OPL	39.7 ± 9.4	39.6 ± 8.9	0.947	38.5 ± 9.0	38.7 ± 8.9	0.912
ONL	107.8 ± 21.4	107.1 ± 23.0	0.748	108.1 ± 22.0	106.8 ± 19.3	0.632

*CMT* = central macular thickness; *NFL* = nerve fiber layer; *GCL* = ganglion cell layer; *IPL* = inner plexiform layer; *INL* = inner nuclear layer; *OPL* = outer plexiform layer; *ONL* = outer nuclear layer

Similarly, in Group 2, the first thickness measurements of CMT, NFL, GCL, IPL, INL, OPL, and ONL were not significantly different from the second measurements.

### Short-term reproducibility of OCT parameters in each group

In Group 1, each retinal layer had generally low reproducibility except for CMT (Table 3).

**Table 3:** Short-term reproducibility of each retinal layer thickness measurement

Parameter	Group 1			Group 2		
	CV (%)	ICC	Reproducibility limit (μm)	CV (%)	ICC	Reproducibility limit (μm)
CMT	1.49	0.964	21.18	1.70	0.962	19.52
NFL	22.39	0.431	47.05	18.20	0.788	17.82
GCL	13.12	0.550	17.43	10.59	0.834	12.17
IPL	13.37	0.440	17.95	10.65	0.830	11.17
INL	13.21	0.286	18.99	13.27	0.715	16.27
OPL	15.09	0.279	16.59	14.75	0.226	15.86
ONL	11.94	0.503	33.28	10.37	0.439	29.99

*CMT* = central macular thickness; *NFL* = nerve fiber layer; *GCL* = ganglion cell layer; *IPL* = inner plexiform layer; *INL* = inner nuclear layer; *OPL* = outer plexiform layer; *ONL* = outer nuclear layer

**Fig. 1:**
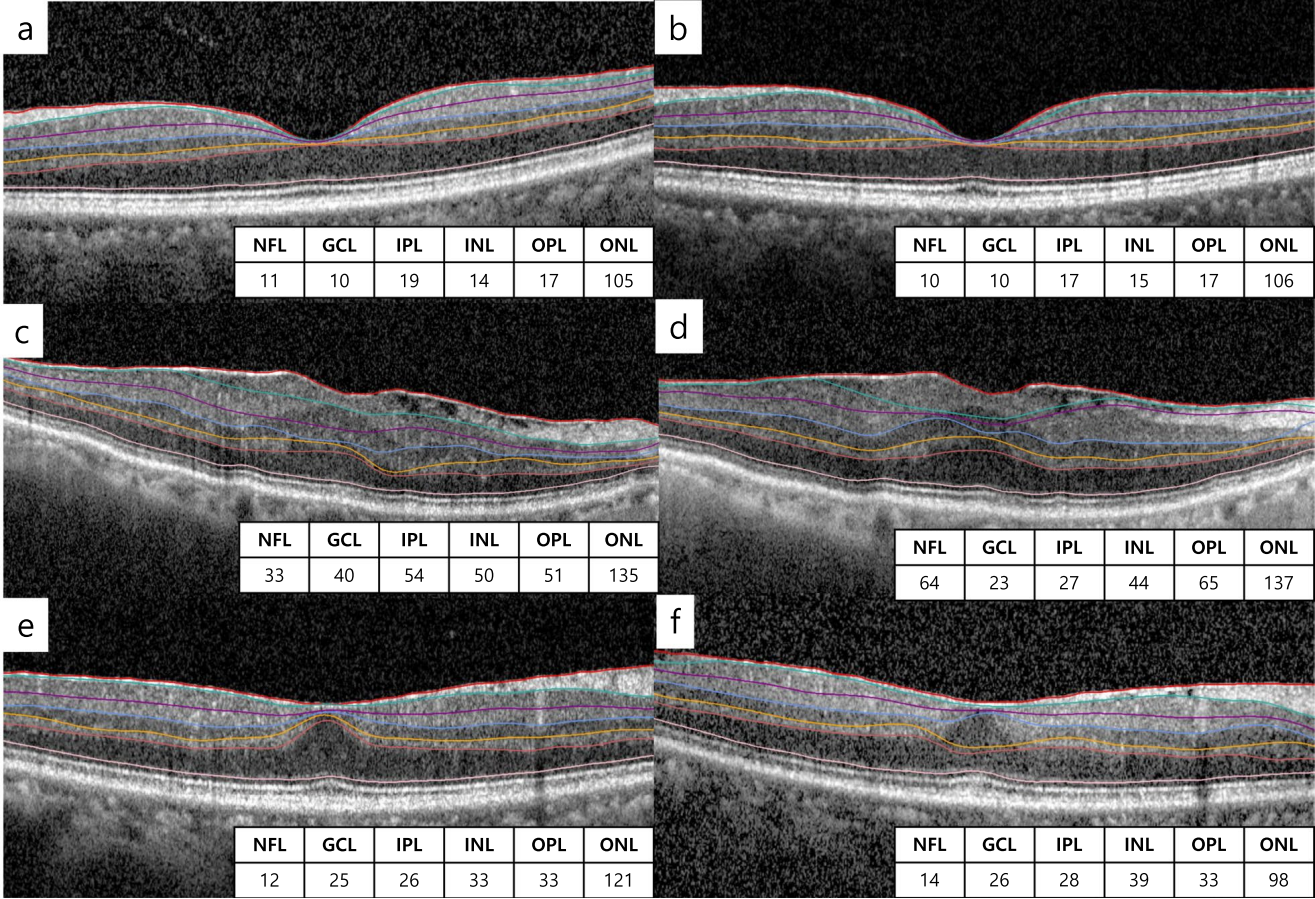
Representative images of consecutive measurements with segmentation and thicknesses (μm) of each layer. Normal contralateral eye (**a**, **b**), patients with ectopic inner foveal layer (**c**, **d**), and patients without ectopic inner foveal layer (**e**, **f**). Most segmentations in (**c**) and (**d**) are different. In (**e**) and (**f**), the segmentation of the inner retinal layer is similar, but the segmentation of the outer retinal layer is different. NFL, nerve fiber layer; GCL, ganglion cell layer; IPL, inner plexiform layer; INL, inner nuclear layer; OPL, outer plexiform layer; ONL, outer nuclear layer

Group 2 showed relatively higher reproducibility compared to Group 1 except for OPL and ONL (Fig. 1).

**Fig. 2:**
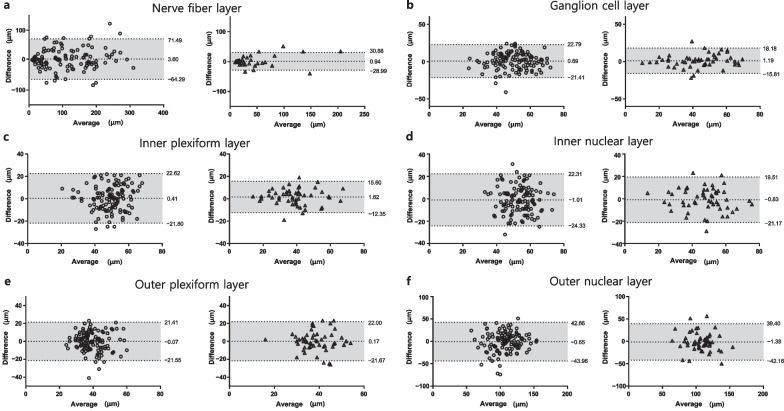
Bland-Altman plots showing the level of agreement for each retinal layer thickness measurement between two consecutive measurements in epiretinal membrane patients with ectopic inner foveal layer (circle) and without ectopic inner foveal layer (triangle). **a** Nerve fiber layer; **b** Ganglion cell layer; **c** Inner plexiform layer; **d** Inner nuclear layer; **e** Outer plexiform layer; **f** Outer nuclear layer

In Bland-Altman plots, Group 1’s measurements, especially NFL, GCL, and IPL, were more scattered compared to Group 2 (Fig. 2).

### Correlation analysis between CMT and the reproducibility of each measurement

**Fig. 3:**
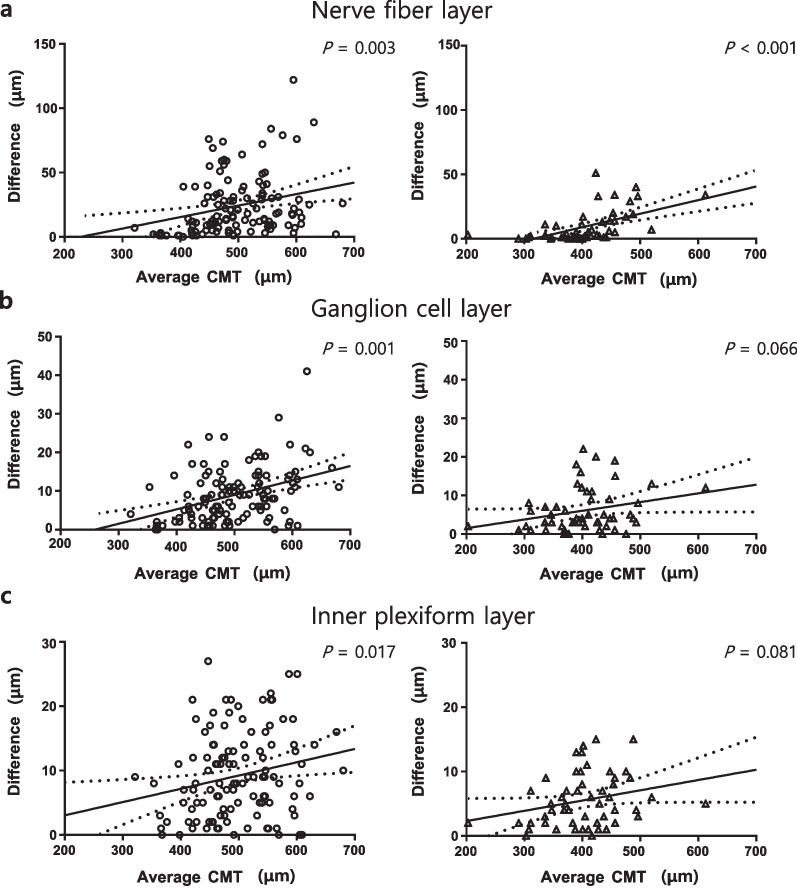
Scatter plots and linear regression analyses between the average central macular thickness (CMT) and inner retinal layers in epiretinal membrane patients with ectopic inner foveal layer (circle) and without ectopic inner foveal layer (triangle). **a** Nerve fiber layer; **b** Ganglion cell layer; **c** Inner plexiform layer

In Group 1, the average CMT was significantly correlated with the CV of CMT (coefficient = 0.183; *P* = 0.046), NFL (coefficient = 0.317; *P* < 0.001), GCL (coefficient = 0.328; *P* < 0.001), and IPL (coefficient = 0.186; *P* = 0.042) (Table 4). In Group 2, only NFL (coefficient = 0.381; *P* = 0.005) showed a significant correlation with the average CMT. Additionally, the differences between the two measurements of NFL (*P* = 0.003), GCL (*P* = 0.001), and IPL (*P* = 0.017) were significantly associated with the average CMT in Group 1, whereas only Group 2’s NFL (*P* < 0.001) showed a significant association (Fig. 3).

**Table 4:** Correlation analysis between average central macular thickness and coefficient of variance values for each retinal layer

Parameter	Group 1		Group 2	
	Coefficient	*P* value	Coefficient	*P* value
CMT	0.183	**0.046**	0.132	0.345
NFL	0.317	** < 0.001**	0.381	**0.005**
GCL	0.328	** < 0.001**	0.059	0.677
IPL	0.186	**0.042**	0.001	0.992
INL	0.165	0.071	− 0.089	0.527
OPL	0.044	0.632	0.020	0.885
ONL	0.016	0.863	0.267	0.054

*CMT* = central macular thickness; *NFL* = nerve fiber layer; *GCL* = ganglion cell layer; *IPL* = inner plexiform layer; *INL* = inner nuclear layer; *OPL* = outer plexiform layer; *ONL* = outer nuclear layer

Bold values indicate statistically significant (*P* < 0.050)

## Discussion

The preoperative thickness of each retinal layer, including the inner retinal layer and ONL, is a significant factor in the postoperative visual prognosis of ERM patients. Thus, accurate measurement of each thickness is crucial. However, studies on the reliability of each retinal layer thickness measurement in ERM patients have been insufficient so far. Furthermore, to the best of our knowledge, there has been no report on how low the reliability of automatic measurement is in ERM patients with EIFLs, compared to those without EIFLs. We found that the measurement of each retinal layer thickness in the foveal area showed low short-term reproducibility in ERM patients with EIFLs. In patients without EIFLs, inner retinal layer thickness measurements had good short-term reproducibility compared to the measurements in patients with EIFLs, but outer retinal layer thicknesses, including OPL and ONL, were less reproducible. Additionally, the short-term reproducibility of the inner retinal layers was significantly correlated with the average CMT in patients with EIFLs, and the difference between the two measurements of the inner retinal layer was significantly associated with the average CMT.

Previous studies have reported good repeatability and reproducibility of CMT measurements using SD-OCT in ERM patients. Lee et al. [18] reported that the ICC and CV of CMT in ERM patients were 0.995 and 0.8%, which was relatively good reproducibility. Pinilla et al. [14] also reported that macular thickness measurements showed a mean CV of 2.95% with a higher ICC than 0.919 using SD-OCT in ERM patients. In our study, the CV and ICC were 1.49% and 0.964 in Group 1, and 1.70% and 0.962 in Group 2, respectively, consistent with previous reports. CMT measurements using SD-OCT in ERM patients are considered highly reliable, regardless of the presence of EIFLs.

On the other hand, most of the retinal layers had low reproducibility in patients with EIFLs, with CVs > 11.00% and ICCs < 0.550. Additionally, the inner retinal layer, including NFL, GCL, and IPL, of patients with EIFLs were more scattered on Bland-Altman plots with larger reproducibility limits compared to patients without EIFLs. Although a previous study reported an ICC of 0.881 for the ganglion cell-inner plexiform layer (GCIPL) in ERM patients, suggesting good reproducibility, this could be attributable to the enrollment of patients without EIFLs, which also showed relatively good reproducibility in our study [18]. In patients with EIFLs, the boundaries of the retinal layers are often tortuous and unclear, and could be blurred and indistinguishable on OCT images, which can cause segmentation errors. Thick EIFLs can be an indication of surgery, but inaccurate measurements by these segmentation errors may interfere with treatment decisions and can be a confounding factor in predicting postoperative prognosis. Therefore, physicians should not rely on automatic retinal layer thickness measurements without first checking the segmentation accuracy of OCT images, and manual adjustments should be performed when obvious segmentation errors are found especially in patients with EIFLs.

Here, the short-term reproducibility of the inner retinal layer thickness measurements in patients without EIFLs was better than in patients with EIFLs. The relatively high CV values of the NFL may be the result of a thinner NFL compared to other retinal layers around the fovea because the CV was calculated as a standard deviation/mean value. Unlike the inner retinal layers’ measurements, which showed relatively good reproducibility, OPL and ONL measurements showed low reproducibility in patients without EIFLs. In patients with advanced ERM without EIFLs, an abnormally wide attachment to the ILM of the ONL and bulging ONL were identified in many OCT images. In these cases, it tends to frequently fail in segmenting the bulged parts well, which could result in unreliable measurements. Apart from the inner retinal layer, the outer retinal layer thickness is also significantly associated with visual function in ERM patients. Arichika et al. [19] reported that outer retinal thickening in the fovea, parafovea, and perifovea was significantly correlated with visual acuity in patients with idiopathic ERM. Cacciamani et al. [20] also found a significant correlation between retinal sensitivity impairment and ONL thickness. Therefore, accurate measurement of the outer retinal layer thickness, including OPL and ONL, is important in ERM patients, and dependence on automatic measurements of outer retinal layer thickness can lead to inaccurate visual prognosis analysis.

Lee et al. [21] reported that the repeatability of GC-IPL thickness measurements was lower in patients with macular edema caused by various retinal diseases including age-related macular degeneration, retinal vein occlusion, central serous chorioretinopathy, and diabetic macular edema. Another study reported low repeatability of GC-IPL measurement in ERM patients with a CMT greater than 450 μm. The proposed explanation was based on frequent auto segmentation errors following the more distorted configuration of the macula, compared with patients with a CMT less than 450 μm [18]. Our study also demonstrated a significant negative correlation between CMT and the short-term reproducibility of inner retinal layer thickness measurements, including NFL, GCL, and IPL, in patients with EIFLs. This was supported by the significant association of the difference between the two measurements of inner retinal layer thickness with the average CMT. However, no significant correlation between the average CMT and reproducibility of GCL and IPL was seen in ERM patients without EIFLs, and the difference between the two measurements was not significantly associated with the average CMT. Therefore, in cases with thick CMT, the importance of manual measurement in patients with EIFLs is more emphasized than in those without EIFL for accurate visual prognosis analysis and adequate explanation for patients because of the low reliability of automatic measurement.

This study has several limitations. First, the retrospective nature of the work inevitably introduced some selection bias. Second, the measurements were acquired with only the Spectralis OCT device; there could be subtle differences among the OCT devices currently in use. Additionally, there are several methods of measuring retinal layer thickness in Spectralis OCT such as a 20° × 20° capture mode with 25 fast scans or the posterior pole algorithm, which can result in varying reproducibility. Fourth, this study demonstrated the short-term reproducibility of OCT in ERM patients with examination intervals of less than one week. However, subtle changes in ERM may occur in a week, and the intervals between the examinations were different among the patients, which could cause some bias. The main strength of this study was that we evaluated the short-term reproducibility of each retinal layer thickness measurement, including the outer retina, in ERM patients, which has rarely been reported. This was also the first study to statistically analyze the short-term reproducibility of each retinal layer thickness measurement in patients with EIFLs and to compare it with that of measurement in patients without EIFLs.

## Conclusions

The short-term reproducibility of the inner retinal layer thickness measurements in ERM patients with EIFLs was lower than in patients without EIFLs, and the short-term reproducibility of the outer retinal layer thickness measurements, including OPL and ONL, was poor, regardless of the presence of EIFLs. Further, the thicker the CMT in patients with EIFLs, the lower the reproducibility of the inner retinal layer thickness measurements and the greater the difference between measurements, whereas patients without EIFLs were less affected by CMT. Physicians should consider the low reliability of outer retinal layer thickness measurements in ERM patients, and inner retinal layer thickness measurements in patients with EIFLs, especially those with severely thickened CMTs. The accuracy of segmentation should be checked, and manual adjustment should be performed if segmentation errors are found.

## Data Availability

The datasets used and/or analyzed during the current study are available from the corresponding author upon reasonable request.
